# Traveling pulses in a stochastic neural field model of direction selectivity

**DOI:** 10.3389/fncom.2012.00090

**Published:** 2012-10-29

**Authors:** Paul C. Bressloff, Jeremy Wilkerson

**Affiliations:** Department of Mathematics, University of UtahSalt Lake City, UT, USA

**Keywords:** stochastic processes, traveling waves, neural field theory, direction selectivity, stimulus-driven

## Abstract

We analyze the effects of extrinsic noise on traveling pulses in a neural field model of direction selectivity. The model consists of a one-dimensional scalar neural field with an asymmetric weight distribution consisting of an offset Mexican hat function. We first show how, in the absence of any noise, the system supports spontaneously propagating traveling pulses that can lock to externally moving stimuli. Using a separation of time-scales and perturbation methods previously developed for stochastic reaction-diffusion equations, we then show how extrinsic noise in the activity variables leads to a diffusive-like displacement (wandering) of the wave from its uniformly translating position at long time-scales, and fluctuations in the wave profile around its instantaneous position at short time-scales. In the case of freely propagating pulses, the wandering is characterized by pure Brownian motion, whereas in the case of stimulus-locked pulses, it is given by an Ornstein–Uhlenbeck process. This establishes that stimulus-locked pulses are more robust to noise.

## Introduction

Continuum neural field models represent the large-scale dynamics of spatially structured networks of neurons in terms of non-linear integro-differential equations, whose associated integral kernels represent the spatial distribution of neuronal synaptic connections (Wilson and Cowan, 1972, 1973; Amari, 1977). As in the case of non-linear partial differential equation (PDE) models of diffusively coupled excitable systems (Keener, 1981; Kuramoto, 1984), non-local neural fields can exhibit a diverse range of spatiotemporal dynamics, including solitary traveling fronts and pulses, stationary pulses, and spatially localized oscillations (breathers), spiral waves, and Turing-like patterns. See, for example, the reviews Ermentrout (1998), Coombes (2005), and Bressloff (2012). In recent years, neural fields have been used to model a wide range of neurobiological phenomena, including wave propagation in cortical slices (Pinto and Ermentrout, 2001; Richardson et al., 2005) and *in vivo* (Huang et al., 2004), geometric visual hallucinations (Ermentrout and Cowan, 1979; Bressloff et al., 2001), EEG rhythms (Nunez, 1995; Robinson et al., 2001; Liley et al., 2002; Steyn-Ross et al., 2003), orientation tuning in primary visual cortex (V1) (Ben-Yishai et al., 1995; Somers et al., 1995), short term working memory (Camperi and Wang, 1998; Laing and Chow, 2002), control of head direction (Zhang, 1996), direction selectivity (Xie and Giese, 2002), motion perception (Giese, 1999), and binocular rivalry waves (Bressloff and Webber, 2012a). One particularly useful feature of neural fields is that analytical techniques for solving these integro-differential equations can be adapted from previous studies of non-linear PDEs. These include regular and singular perturbation methods, weakly non-linear analysis and pattern formation, symmetric bifurcation theory, Evans functions and wave stability, and homogenization theory (Bressloff, 2012). In particular, we have recently shown how perturbation methods for studying fluctuating fronts in reaction-diffusion PDEs (Schimansky-Geier et al., 1983; de Pasquale et al., 1992; Armero et al., 1998; Sagues et al., 2007) can be extended to the problem of front propagation in stochastic neural fields (Bressloff and Webber, 2012b), and have used this to investigate the effects of noise on binocular rivalry waves (Webber and Bressloff, submitted). Such methods exploit a separation of time-scales in which there is a diffusive-like displacement (wandering) of the front from its uniformly translating position at long time-scales, and fluctuations in the front profile around its instantaneous position at short time-scales.

In this paper, we extend our theory of wave propagation in stochastic neural fields to the case of a neural field that supports traveling pulses rather than fronts. A typical mechanism for generating traveling pulses in an excitatory network is to include some form of slow adaptation, such as spike frequency adaptation (Pinto and Ermentrout, 2001; Coombes and Owen, 2005) or synaptic depression (Kilpatrick and Bressloff, 2010a,b), which suppresses the trailing edge of the wave. One of the motivations for considering excitatory neural fields is that traveling pulses are observed in *in vitro* cortical slices that have been disinhibited. Here we consider an alternative mechanism for generating pulses, based on asymmetric excitatory/inhibitory synaptic connections. Such a network architecture supports freely propagating pulses without any adaptation, and has been proposed as a simple recurrent mechanism for generating direction selectivity in a network driven by moving stimuli (Mineiro and Zipser, 1998; Xie and Giese, 2002). Most classical models for the direction selectivity of cortical neurons are based on feedforward mechanisms, namely, the linear or non-linear spatiotemporal filtering of afferent thalamo-cortical inputs (Reichardt, 1961; Adelson and Bergen, 1985; Koch and Poggio, 1985; van Santen and Sperling, 1985). Some of these models also involve a combination of lagged (time-delayed) and non-lagged inputs (Saul and Humphrey, 1990; Baker and Bair, 2012). However, there is now considerable experimental data demonstrating that the response of cortical cells is strongly influenced by intracortical circuitry. This has motivated a number of modeling studies that show how direction selectivity can be reproduced by recurrent neural network models with asymmetric lateral excitatory or inhibitory connections and non-direction-selective inputs (Suarez et al., 1995; Maex and Urban, 1996; Mineiro and Zipser, 1998; Xie and Giese, 2002). In this paper, we base our investigation of stochastic traveling pulses on the particular version introduced by Xie and Giese (2002).

The main results of the paper are as follows. We first analyze freely propagating pulses and stimulus-locked pulses in the deterministic case, expanding the analysis of Xie and Giese (2002). In particular, we construct a stability diagram showing the existence and stability of stimulus-locked pulses as a function of stimulus velocity and amplitude. We then turn to a corresponding stochastic version of the model. We show how extrinsic noise in the activity variable leads to a diffusive-like displacement (wandering) of the wave from its uniformly translating position at long time-scales, and fluctuations in the wave profile around its instantaneous position at short time-scales. In the case of freely propagating pulses, the wandering is characterized by pure Brownian motion, whereas in the case of stimulus-locked pulses, it is given by an Ornstein–Uhlenbeck process. This establishes that stimulus-locked pulses are more robust to noise. One major difference between pulses and fronts is that, in principle, noise could significantly affect both the location (center-of-mass) and width of the pulse. We find that fluctuations in the width can be neglected in the case of freely propagating pulses, whereas the saturation of the mean-square displacement of the center-of-mass of the pulse for stimulus-locked pulses means that fluctuations in pulse width can no longer be ignored.

## Materials and methods

### Neural field model of direction selectivity

In this paper we consider a scalar neural field equation of the form

(1) $$\tau \frac{\partial u(x,t)}{\partial t}=-u(x,t)+\int_{-\infty }^{\infty }w(x-x^{\prime })F(u(x^{\prime },t))dx^{\prime }+h(x,t)$$

Here *u*(*x*, *t*) is a measure of activity (current or voltage) within a local population of excitatory and inhibitory neurons at position *x* ∈ ℝ and time *t*, τ is a membrane time constant (of order 10 msec), *w*(*x*) denotes the spatial distribution of synaptic connections between local populations, *F*(*u*) is a non-linear firing rate function and *h*(*x*, *t*) is an external input. (We fix the time-scale by setting τ = 1). *F* is usually taken to be a sigmoid function

(2) $$F(u)=\frac{1}{1+e^{-\gamma (u-\kappa )}}$$

with gain γ and threshold κ. In the high-gain limit γ → ∞, this reduces to the Heaviside function

(3) $$F(u)\to H(u-\kappa )=\{\begin{array}{cc} 1 & \;if\;u>\kappa \\ 0 & \;if\;u\leq \kappa . \end{array}$$

**Figure 1:**
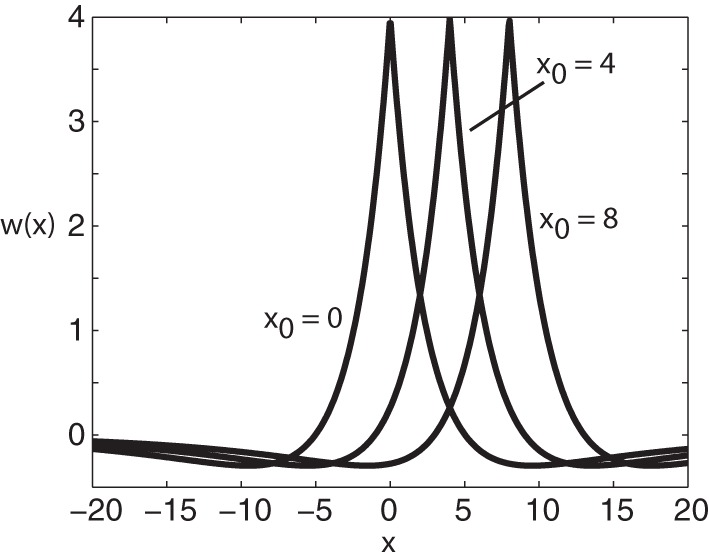
**Plot of weight distribution for various values of the shift *x*_0_.** Here *a*_*e*_ = 5, *a*_i_ = 1, σ_*e*_ = 0.42, and σ_i_ = 0.1.

The function *w*(*x* − *x*') represents the distribution of synaptic weights from the local population at *x*' to the population at *x*. Usually, *w* is taken to be a symmetric or even function such that *w*(*x*) = w(−x). A common choice for the weight distribution is a “Mexican hat” function, with a center excitatory region surrounded by flanking inhibitory regions. As originally shown by Amari (1977), symmetric Mexican hat functions tend to support stationary activity “bumps.” Following Xie and Giese (2002), however, we will use an asymmetric Mexican hat function whose maximum is offset by an amount *x*_0_, that is *w*(*x* − *x*_0_) = w(−[*x* − *x*_0_]); the resulting neural field then supports freely propagating pulses that depend on the degree of offset. Note that such a choice should be contrasted with a symmetric function *w* with peaks offset from zero see e.g., (Hutt and Atay, 2005). In the case of exponential functions, *w* takes the form (see Figure 1)

(4) $$w(x)=a_{e}{\text{e}}^{-\sigma_{e}|x-x_{0}|}-a_{i}{\text{e}}^{-\sigma_{i}|x-x_{0}|},$$

where *a*_*e*_ > a_*i*_ and σ_*e*_ > σ_*i*_. Setting *x*_0_ = 0 recovers the standard Mexican hat function. Note that one could equally use other functions such as a difference-of-Gaussians without changing the main results of the paper; the advantage of exponentials is that one can carry out explicit calculations.

Finally, the external input *h*(*x*, *t*) consists of two components:

(5) $$h(x,t)=I(x-vt)+\sqrt{\epsilon }g(u(x,t))\xi (x,t).$$

Here *I*(*x* − *vt*) represents an external pulse-like stimulus moving with constant speed *v* and amplitude *I*_0_, whereas the second term represents an extrinsic, multiplicative noise source. In particular, ξ(*x*, *t*) is a Gaussian process with zero mean and two-point correlations

(6) $$\langle \eta (x,t)\eta (x^{\prime },t^{\prime })\rangle =2C(|x-x^{\prime }|/\lambda )\delta (t-t^{\prime }).$$

Thus the noise is white in time and colored in space with correlation length λ. Formally speaking, η(*x*, *t*)*dt* = *dW*(*x*, *t*) where *dW*(*x*, *t*) is a corresponding Wiener process.The amplitude of the noise is determined by the parameter ∈, and the function *g*(*u*) incorporates any activity-dependence. Note that (Xie and Giese, 2002) only considered the deterministic case (∈ = 0). They showed how the deterministic neural field supports freely propagating pulses of fixed speed *c* when *I*_0_ = 0. This then provides a mechanism for direction selectivity, since these pulses can lock to a moving stimulus of speed *v* provided that |*c* − *v*| is sufficiently small; the range of locking depends on the amplitude *I*_0_. In this paper, we develop a more systematic analysis of stimulus-locking in the absence of noise, and then investigate the effects of noise on both freely propagating and stimulus-locked pulses.

## Results

### Determinstic neural field

We begin by analyzing traveling pulse solutions of the neural field Equation (1) in the absence of noise (∈ = 0). Following the original formulation of Amari (1977), we investigate the existence and stability of traveling pulses by setting the firing rate function to be the Heaviside (Equation 3).

#### Freely propagating pulses

For the moment, suppose that there are no external inputs so that *h*(*x*, *t*) = 0 in Equation (1). A traveling pulse of velocity *c* is then defined according to *u*(*x*, *t*) = *U*(ξ), with ξ = *x* − *ct* a traveling wave coordinate such that lim_ξ→±∞_ *U*(ξ) = 0. Moreover, the wave profile is restricted to be super threshold in a connected interval of width *d*. Since the neural field is equivariant with respect to uniform translations (in the absence of external stimuli), we choose the two threshold crossing points to be

(7) U(0)=κ, U(d)=κ.

Thus, *U*(ξ) > κ for 0 < ξ < *d*, *U*(ξ) < κ for ξ < 0, and ξ > *d*. It turns out the wave travels in the same direction as the offset so we restrict ourselves to the case *x*_0_ > 0 and *c* > 0. Substituting the traveling pulse solution into Equation (1) gives

(8) $$-c\frac{\partial U(\xi )}{\partial \xi }=-U(\xi )+\int_{0}^{d}w(\xi -\xi^{\prime })d\xi^{\prime }$$

Multiplying both sides by e^−ξ/c^ and integrating gives the following equation for the wave solution:

(9) $$U(\xi )=\frac{e^{\xi /c}}{c}\int_{\xi }^{\infty }\text{​}W(\xi^{\prime }){\text{e}}^{-\xi^{\prime }/c}\text{d}\xi^{\prime },$$

where

$$W(\xi )\equiv \int_{\xi -d}^{\xi }\text{​}w(x)\text{d}x.$$

It is convenient to express the weight function in piecewise form as follows:

(10) $$w(x)=\{\begin{array}{ll} a_{e}{\text{e}}^{-\sigma_{e}(x-x_{0})}-a_{i}{\text{e}}^{-\sigma_{i}(x-x_{0})}\equiv w_{1}(x), & \text{if }x\geq x_{0}\\ a_{e}{\text{e}}^{\sigma_{e}(x-x_{0})}-a_{i}{\text{e}}^{\sigma_{i}(x-x_{0})}\equiv w_{2}(x), & \text{if }x\leq x_{0} \end{array}$$

We then obtain a piecewise expression for *W*(ξ) of the form

(11) $$W(\xi )=\{\begin{array}{ll} W_{3}(\xi )\equiv \int_{\xi -d}^{\xi }\text{​}w_{2}(x)\text{d}x, & \text{if }\xi \leq x_{0}\\ W_{2}(\xi )\equiv \int_{\xi -d}^{x_{0}}\text{​}w_{2}(x)\text{d}x & \\ \;+\int_{x_{0}}^{\xi }\text{​}w_{1}(x)\text{d}x, & \text{if }x_{0}\leq \xi \leq x_{0}+d\\ W_{1}(\xi )\equiv \int_{\xi -d}^{\xi }\text{​}w_{1}(x)\text{d}x, & \text{if }\xi \geq x_{0}+d \end{array}$$

We then have

$$U(\xi )=\{\begin{array}{ll} \frac{1}{c}{\text{e}}^{\xi /c}(M_{3}(\xi )+M_{1}(x_{0}+d) & \\ \;+M_{2}(x_{0})), & \text{if }\xi \leq x_{0}\\ \frac{1}{c}{\text{e}}^{\xi /c}\left(M_{2}(\xi )+M_{1}(x_{0}+d)\right), & \text{if }x_{0}\leq \xi \leq x_{0}+d\\ \frac{1}{c}{\text{e}}^{\xi /c}M_{1}(\xi ), & \text{if }\xi \geq x_{0}+d \end{array}.$$

where

(12) $$M_{n}(\xi )=\int_{\xi }^{\xi_{n}}W_{n}(\xi^{\prime }){\text{e}}^{-\xi^{\prime }/c}d\xi^{\prime }$$

with ξ_1_ = ∞, ξ_2_ = *x*_0_ + *d* and ξ_3_ = *x*_0_.

**Figure 2:**
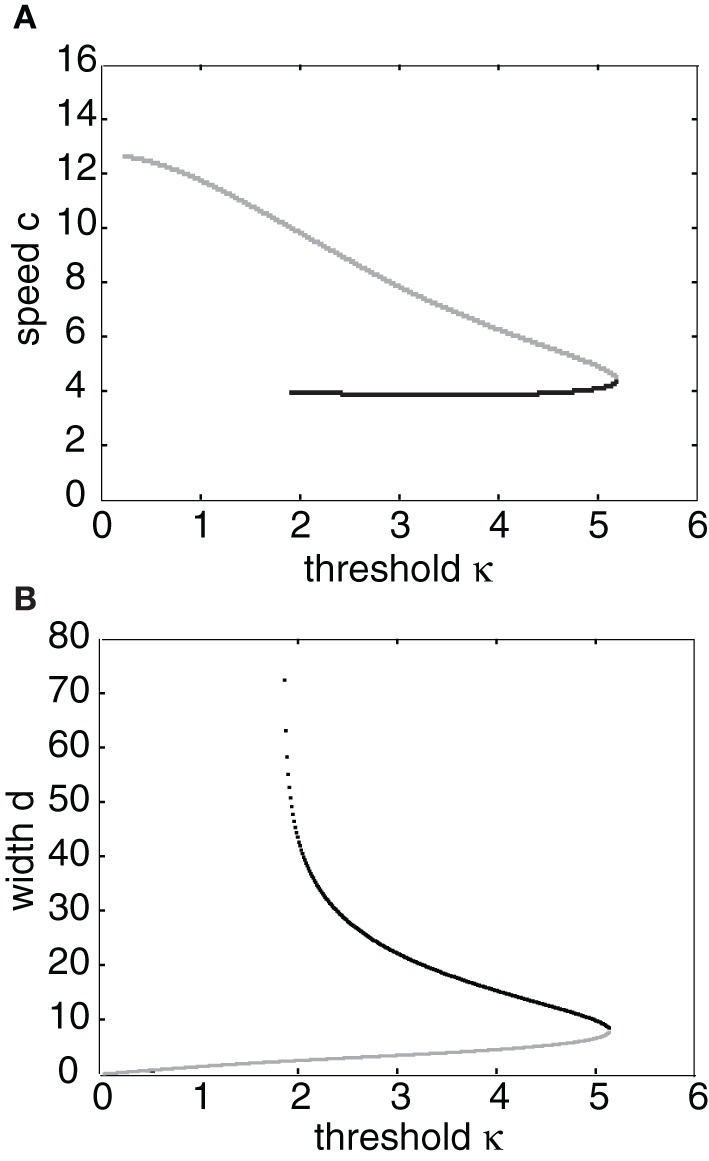
**(A)** Plots of pulse speed ***c*** and **(B)** pulse width ***d*** as a function of the threshold κ. Weight parameters are as in Figure 1 with offset *x*_0_ = 3. Stable (unstable) branches are indicated by black (gray) curves.

**Figure 3:**
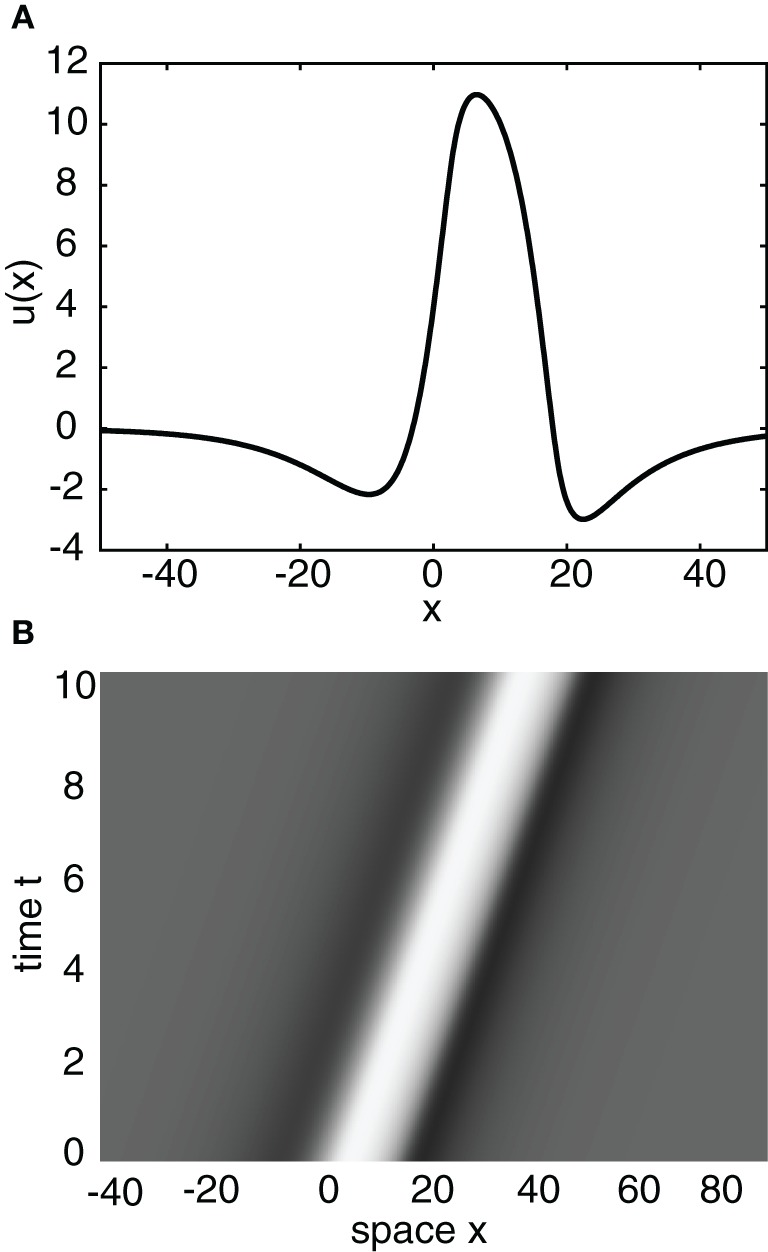
**(A)** Plot of traveling wave profile *U*(ξ) obtained analytically. Same parameters as Figure 2 for threshold κ = 4. **(B)** Spacetime plot of a traveling pulse using the profile of **(A)** as the initial condition. High (low) activity indicated by light (gray).

Having obtained the piecewise wave profile *U*(ξ), the threshold conditions (Equation 7) can now be used to determine the pulse speed *c* and width *d*; the resulting transcendental equations have to be solved numerically. Figure 2 shows solutions for the pulse speed and width as functions of the threshold. It turns out that the solution with slower speed (and larger width) is stable (see below). This differs from traveling pulse solutions found in adaptive neural fields, where the faster wave (with larger width) tends to be stable (Pinto and Ermentrout, 2001; Kilpatrick and Bressloff, 2010a). Figure 3A shows a typical pulse waveform and Figure 3B shows a numerical simulation of the neural field Equation (1) using the wave solution as the initial condition. The pulse propagates at the predicted speed without changing shape significantly. This occurs because the parameters were chosen to make the pulse solution linearly stable.

#### Stability

In order to determine the linear stability of a traveling pulse solution *U*(ξ) in the moving frame, we linearize Equation (1) with *h*(*x*, *t*) = 0 by setting

U(ξ, t)=U(ξ)+φ(ξ, t),

and Taylor expanding to first order in ϕ. This gives

(13) $$\frac{\partial \varphi (\xi ,t)}{\partial t}=c\frac{\partial \varphi (\xi ,t)}{\partial \xi }-\varphi (\xi ,t)+\int_{-\infty }^{\infty }\text{​}w(\xi -y)\times F^{\prime }(U(y))\varphi (y,t)\text{d}y.$$

In the case of the Heaviside rate function (Equation 3), we have

(14) $$F^{\prime }(U(\xi ))=\frac{\delta (\xi )}{\left|U^{\prime }(0)\right|}+\frac{\delta (\xi -d)}{\left|U^{\prime }(d)\right|}.$$

Moreover, differentiating Equation (9) with respect to ξ shows that

$$U^{\prime }(\xi )=\frac{1}{c}(U(\xi )-W(\xi )).$$

Substituting the previous two results into Equation (13) gives

(15) $$\begin{array}{l} \frac{\partial \varphi (\xi ,\;t)}{\partial t}=ℒ\varphi (\xi ,\;t)\\ \;\equiv c\frac{\partial \varphi (\xi ,\;t)}{\partial \xi }-\varphi (\xi ,\;t)+\frac{c\varphi (0,\;t)}{\left|\kappa -W(0)\right|}w(\xi )+\frac{c\varphi (d,\;t)}{\left|\kappa -W(d)\right|}w(\xi -d). \end{array}$$

where κ is the threshold. Looking for solutions of the form

(16) $$\varphi (\xi ,t)={\text{e}}^{\lambda t}\varphi (\xi ).$$

then leads to the spectral problem

(17) ℒφ(ξ, t)=λφ(ξ, t).

We take the linear operator ℒ to act on a Banach space ℬ of continuous, bounded functions ψ(ξ) that are defined for ξ ∈ ℝ, and that decay exponentially as ξ→±∞. Let σ(ℒ) denote the spectrum of the linear operator ℒ, and define the associated resolvent operator according to ℛ_λ_ ≡ (ℒ − λ *I*)^−1^, where *I* is the identity operator. The spectrum can be defined as those values of λ for which $T_{\lambda }\equiv ℒ-\lambda I$ is not bijective. The spectrum is composed of three disjoint sets, the point or discrete spectrum, the residual spectrum, and the continuous spectrum. The point spectrum is defined as the values of λ (eigenvalues) for which the resolvent does not exist. The residual spectrum are the spectral values for which the resolvent exists but is not defined on a dense subset of ℬ. The continuous spectrum are the spectral values for which the resolvent exists and is densely defined but is unbounded (Kreyszig, 1978). Given these definitions, the traveling pulse is said to be linearly stable if (1) Re(λ) < 0 for all λ ın σ(ℒ), λ ≠ 0 and (2) the zero eigenvalue is simple. The existence of a zero eigenvalue with corresponding eigenfunction ϕ(ξ, *t*) = *U*′(ξ) reflects translation invariance, and immediately follows from differentiating (Equation 9) with respect to ξ.

We first consider the discrete spectrum by solving the eigenvalue equation

$$\frac{d\psi (\xi )}{d\xi }-\frac{\lambda +1}{c}\psi (\xi )+K_{0}\psi (0)w(\xi )+K_{d}\psi (d)w(\xi -d)=0,$$

where we have introduced the constants

$$K_{0}=\frac{1}{\left|\kappa -W(0)\right|}\text{ and }K_{d}=\frac{1}{\left|\kappa -W(d)\right|}.$$

Multiplying both sides by e^−(λ+1)ξ/c^ and integrating gives

$$\psi (\xi )=K_{0}\psi (0)\int_{\xi }^{\infty }\text{​}w(y){\text{e}}^{(\lambda +1)(\xi -y)/c}\text{d}y+K_{d}\psi (d)\int_{\xi -d}^{\infty }\text{​}w(y){\text{e}}^{(\lambda +1)(\xi -d-y)/c}\text{d}y,$$

which can be rewritten in the more compact form

(18) $$\psi (\xi )=K_{0}\psi (0)(w\times P_{\lambda })(\xi )+K_{d}\psi (d)(w\times P_{\lambda })(\xi -d)$$

with

(19) $$(w\times P_{\lambda })(\xi )=\int_{-\infty }^{\infty }\text{​}w(y)P_{\lambda }(\xi -y)\text{d}y,\;P_{\lambda }(\xi )=H(-\xi ){\text{e}}^{(\lambda +1)\xi /c}$$

The eigenvalues are now determined by imposing self-consistency at ξ = 0 and ξ = *d*. Setting ξ = 0 and ξ = *d* in Equation (18) leads to the vector equation

(20) $$\begin{array}{c} \left[\begin{array}{ll} K_{0}(w\times P_{\lambda })(0)-1 & K_{d}(w\times P_{\lambda })(-d)\\ K_{0}(w\times P_{\lambda })(d) & K_{d}(w\times P_{\lambda })(0)-1 \end{array}\right]\left[\begin{array}{l} \psi (0)\\ \psi (d) \end{array}\right]=0 \end{array}$$

This has a non-trivial solution if and only if the determinant of the matrix is zero. The determinant expressed as a function of λ, ℰ(λ), is a complex analytic function known as the Evans function:

(21) $$ℰ(\lambda )=\left[K_{0}(w\times P_{\lambda })(0)-1\right]\left[K_{d}(w\times P_{\lambda })(0)-1\right]-K_{0}K_{d}(w\times P_{\lambda })(d)(w\times P_{\lambda })(-d).$$

**Figure 4:**
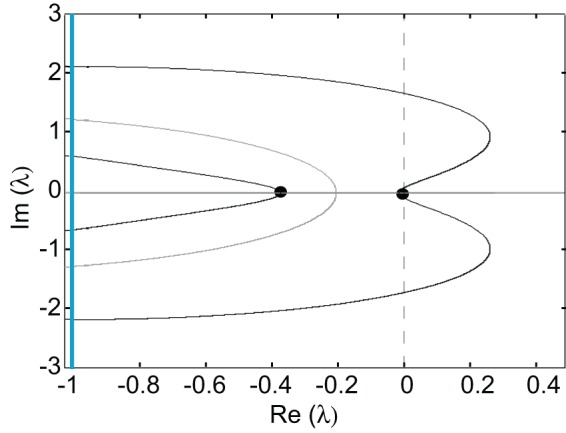
**Graphs of the zero sets of the real (*dark curves*) and imaginary (*light curves*) parts of the Evans function determining the stability of a freely propagating pulse; intersection points (filled circles) indicate eigenvalues.** The line *I*m λ = −1 indicates the essential spectrum. Same parameter values as Figure 3.

Thus, the zeros of the Evans function determine the discrete spectrum of the linear operator formed by linearizing the neural field equation about the pulse solution. Evans functions were originally introduced within the context of the stability of solitary pulses in diffusive Hodgkin–Huxley type equations describing action potential propagation in nerve axons (Evans, 1975). Since then the Evans function construction has been extended to a wide range of PDEs, see the review (Sandstede, 2002). It has also recently been applied to neural field equations (Zhang, 2003; Coombes and Owen, 2004; Rubin, 2004; Folias and Bressloff, 2005; Pinto et al., 2005; Sandstede, 2007) and more general non-local problems (Kapitula et al., 2004). An example plot of the real and imaginary parts of ℰ(λ) = 0 on the complex plane is shown in Figure 4. It can be seen that there is a zero eigenvalue and one negative real eigenvalue, indicating that the corresponding traveling pulse is linearly stable.

To find the essential spectrum, which is the union of the residual and continuous spectra, we will derive an explicit expression for the resolvent ℛ_λ_. We start by writing an inhomogeneous equation of the form $T_{\lambda }\psi (\xi )=h(\xi ),$ where *h*(ξ) represents a general function from the Banach space ℬ. This can be manipulated as before to give

(22) $$\psi (\xi )=-(h\times P_{\lambda })(\xi )+K_{0}\psi (0)(w\times P_{\lambda })(\xi )+K_{d}\psi (d)(w\times P_{\lambda })(\xi -d)$$

By evaluating at ξ = 0 and ξ = *d* as before, we arrive at the following vector equation, which differs from (Equation 20) only on the right-hand side:

$$\begin{array}{l} \left[\begin{array}{ll} K_{0}(w\times P_{\lambda })(0)-1 & K_{d}(w\times P_{\lambda })(-d)\\ K_{0}(w\times P_{\lambda })(d) & K_{d}(w\times P_{\lambda })(0)-1 \end{array}\right]\left[\begin{array}{l} \psi (0)\\ \psi (d) \end{array}\right]\\ \;=\left[\begin{array}{l} \left(h\times P_{\lambda })(0\right)\\ \left(h\times P_{\lambda })(d\right) \end{array}\right]. \end{array}$$

Since we are looking for spectral values outside the discrete spectrum, the determinant of the matrix satisfies ℰ(λ)≠ 0. Therefore, multiplying both sides by the inverse matrix yields expressions for ψ(0) and ψ(*d*) in terms of *h*:

$$\psi (0)=\frac{S_{0}h}{ℰ(\lambda )},\;\psi (d)=\frac{S_{d}h}{ℰ(\lambda )}$$

where

$$S_{0}h=\left(K_{d}(w\times P_{\lambda })(0)-1\right)(h\times P_{\lambda })(0)-K_{d}(w\times P_{\lambda })(-d)(h\times P_{\lambda })(d)$$

and

$$S_{d}h=-K_{0}(w\times P_{\lambda })(d)(h\times P_{\lambda })(0)+\left(K_{0}(w\times P_{\lambda })(0)-1\right)(h\times P_{\lambda })(d).$$

Substituting into Equation (22) gives the following expression for the resolvent operator, of the form ℛ_λ_*h* = ϕ:

(23) $$-(h\times P_{\lambda })(\xi )+\frac{K_{0}S_{0}h}{ℰ(\lambda )}(w\times P_{\lambda })(\xi )+\frac{K_{d}S_{d}h}{ℰ(\lambda )}(w\times P_{\lambda })(\xi -d)=\psi (\xi )$$

The resolvent is well-defined for all *h* in ℬ, so the residual spectrum of ℒ is empty. To find the continuous spectrum, we Fourier transform Equation (23):

(24) $$-\hat{h}(k){\hat{P}}_{\lambda }(k)+\frac{K_{0}S_{0}h}{\varepsilon (\lambda )}\hat{w}(k){\hat{P}}_{\lambda }(k)+\frac{K_{d}S_{d}h}{\varepsilon (\lambda )}\hat{w}(k){\hat{P}}_{\lambda }(k){\text{e}}^{-2\pi idk}=\hat{\psi }(k)$$

It follows that the resolvent operator is unbounded when ${\hat{P}}_{\lambda }$ is unbounded. Equation (19) implies that

(25) $${\hat{P}}_{\lambda }(k)=\int_{-\infty }^{\infty }\text{​}P_{\lambda }(\xi ){\text{e}}^{2\pi ik\xi }\text{ d}\xi =\frac{1}{\frac{\lambda +1}{c}+2\pi ik}.$$

Hence, ${\hat{P}}_{\lambda }$ is unbounded for λ = −1 − 2π*ikc* so that the continuous spectrum of ℒ is a vertical line in the complex plane at Re(λ) = −1. Since Re(λ) < 0, the continuous spectrum will not make any pulse solution of our model unstable.

#### Stimulus-locked pulses

Now suppose that the neural field is driven by a moving external pulse stimulus of speed *v* so that Equation (1) becomes

(26) $$\frac{\partial u(x,t)}{\partial t}=-u(x,t)+\int_{-\infty }^{\infty }\text{​}w\left(x-y\right)H\left(u\left(y,t\right)-\kappa \right)\text{d}y+\;I(x-vt).$$

In order to study the existence of stimulus-locked pulses, we will define a “stimulus coordinate” ξ = *x* − *vt* and look for pulse solutions that move at the same speed as the stimulus, that is, *u*(*x*, *t*) = *U*(ξ) with

(27) $$-v\frac{\partial U(\xi )}{\partial \xi }=-U(\xi )+\int_{-\infty }^{\infty }\text{​}w(\xi -y)H(U(y)-\kappa )\text{d}y+I(\xi ).$$

For concreteness, the stimulus will be represented by a rectangular wave of amplitude *I*_0_ and width *d*, defined formally as

$$I(\xi )=\{\begin{array}{ll} I_{0}, & \text{if }0\leq \xi \leq d\\ 0, & \text{if }\xi <0\text{ or }\xi >d. \end{array}$$

Since translation invariance no longer holds, it is necessary to determine both threshold crossing points, which we denote by ξ = *d*_1_ and ξ = *d*_2_. Proceeding in a similar fashion to the case of freely propagating pulses, we find that

$$U(\xi )=\frac{{\text{e}}^{\xi /v}}{v}\int_{\xi }^{z_{0}}\text{​}{\text{e}}^{-y/v}W(y)\text{ d}y+\frac{{\text{e}}^{\xi /v}}{v}\int_{\xi }^{z_{0}}\text{​}{\text{e}}^{-y/v}I(y)\text{ d}y,$$

where *z*_0_ = ∞ if *v* > 0, *z*_0_ = −∞ if *v* < 0, and

$$W(\xi )\equiv \int_{\xi -d_{2}}^{\xi -d_{1}}\text{​}w(x)\text{ d}x.$$

The latter can be expressed in the piecewise form

(28) $$W(\xi )=\{\begin{array}{ll} W_{3}(\xi )\equiv \int_{\xi -d_{2}}^{\xi -d_{1}}\text{​}w_{2}(x)\text{ d}x, & \text{if }\xi \leq x_{0}+d_{1}\\ W_{2}(\xi )\equiv \int_{\xi -d_{2}}^{x_{0}}\text{​}w_{2}(x)\text{ d}x & \\ +\int_{x_{0}}^{\xi -d_{1}}\text{​}w_{1}(x)\text{ d}x, & \text{if }x_{0}+d_{1}\leq \xi \leq x_{0}+d_{2}\\ W_{1}(\xi )=\int_{\xi -d_{2}}^{\xi -d_{1}}\text{​}w_{1}(x)\text{ d}x, & \text{if }\xi \geq x_{0}+d_{2} \end{array}$$

where *w*_1_ and *w*_2_ are defined as in Equation (10). After evaluating the integrals along similar lines to section “Neural Field Model of Direction Selectivity,” we obtain the following expressions for the pulse solution, defined independently for positive and negative stimulus directions:

*v* > 0::

$$U(\xi )=\{\begin{array}{ll} U_{3}(\xi ), & \text{if }\xi \leq x_{0}+d_{1}\\ U_{2}(\xi ), & \text{if }x_{0}+d_{1}\leq \xi \leq x_{0}+d_{2},\\ U_{1}(\xi ), & \text{if }\xi \geq x_{0}+d_{2}, \end{array}$$

with

$$\begin{array}{l} U_{3}(\xi )=\frac{1}{v}{\text{e}}^{\xi /v}\left(M_{3}(\xi )+M_{1}(x_{0}+d_{2})+M_{2}(x_{0}+d_{1})\right)+Z(\xi )\\ U_{2}(\xi )=\frac{1}{v}{\text{e}}^{\xi /v}\left(M_{2}(\xi )+M_{1}(x_{0}+d_{2})\right)+Z(\xi )\\ U_{1}(\xi )=\frac{1}{v}{\text{e}}^{\xi /v}M_{1}(\xi )+Z(\xi ),\\ M_{n}(\xi )=\int_{\xi }^{\xi_{n}}W_{n}(\xi^{\prime }){\text{e}}^{-\xi^{\prime }/v}d\xi^{\prime } \end{array}$$

for ξ_1_ = ∞, ξ_2_ = *x*_0_ + *d*_2_, ξ_3_ = *x*_0_ + *d*_1_, and

$$Z(\xi )=\{\begin{array}{ll} \left({\text{e}}^{\xi /v}-{\text{e}}^{(\xi -d)/v}\right)I_{0}, & \text{if }\xi <0\\ \left(1-{\text{e}}^{(\xi -d)/v}\right)I_{0}, & \text{if }0\leq \xi \leq d.\\ 0, & \text{if }\xi >d \end{array}$$

*v* < 0:

$$U(\xi )=\{\begin{array}{ll} U_{3}(\xi ), & \text{if }\xi \leq x_{0}+d_{1}\\ U_{2}(\xi ), & \text{if }x_{0}+d_{1}\leq \xi \leq x_{0}+d_{2},\\ U_{1}(\xi ), & \text{if }\xi \geq x_{0}+d_{2} \end{array}$$

with

$$\begin{array}{l} U_{3}(\xi )=-\frac{1}{v}{\text{e}}^{\xi /v}N_{3}(\xi )+Z(\xi )\\ U_{2}(\xi )=-\frac{1}{v}{\text{e}}^{\xi /v}\left(N_{2}(\xi )+N_{3}(x_{0}+d_{1})\right)+Z(\xi )\\ U_{1}(\xi )=-\frac{1}{v}{\text{e}}^{\xi /v}\left(N_{1}(\xi )+N_{2}(x_{0}+d_{1})+N_{3}(x_{0}+d_{1})\right)+Z(\xi )\\ N_{n}(\xi )=\int_{\xi_{n}}^{\xi }W_{n}(\xi^{\prime }){\text{e}}^{-\xi^{\prime }/v}d\xi^{\prime } \end{array}$$

for ξ_3_ = −∞, ξ_2_ = *x*_0_ + *d*_1_, ξ_1_ = *x*_0_ + *d*_2_, and

$$Z(\xi )=\{\begin{array}{ll} 0, & \text{if }\xi <0\\ \left(1-{\text{e}}^{\xi /v}\right)I_{0}, & \text{if }0\leq \xi \leq d.\\ \left({\text{e}}^{(\xi -d)/v}-{\text{e}}^{\xi /v}\right)I_{0}, & \text{if }\xi >d \end{array}$$

**Figure 5:**
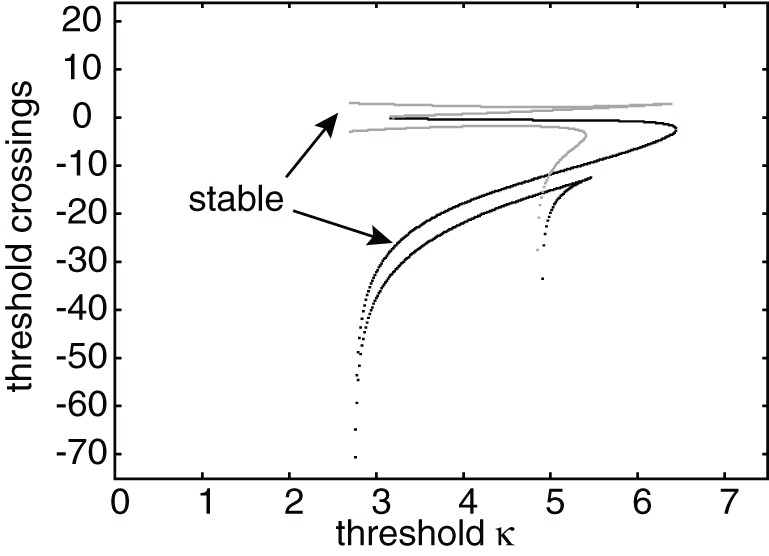
**Plot of leading threshold crossing position *d*_2_ (gray curves) and trailing threshold crossing position *d*_1_ (black curves) of stimulus-locked pulses as a function of threshold κ.** There exists at most one stable pulse (indicated by arrows) and up to three unstable pulses. Weight parameters as in Figure 2 and κ = 4. Stimulus parameters are *d* = 5, *I*_0_ = 5, and *v* = 5.

**Figure 6:**
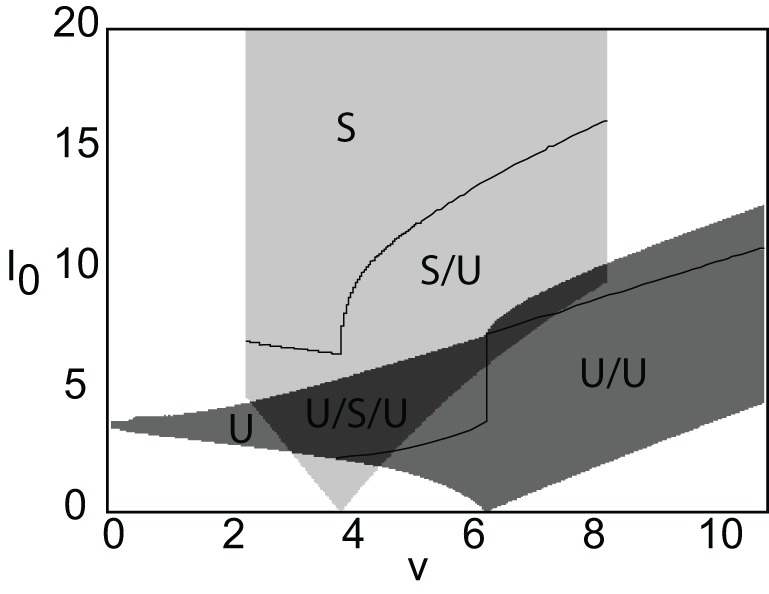
**Stability diagram for stimulus-locked pulses in (*v*, *I*_0_)-parameter space.** Weight parameters as in Figure 2, κ = 4, and *d* = 5. Emerging from the stable pulse solution when *I*_0_ = 0 is a tongue consisting of a stable/unstable pair of pulses (light gray). Similarly, emerging from the unstable solution when *I*_0_ = 0 is a tongue consisting of two unstable pulses (medium gray). As *I*_0_ increases within a tongue an unstable pulse can disappear due to the development of multiple super-threshold regions (indicated by solid curves). All solutions coexist when tongues overlap (dark gray).

The threshold crossing points (*d*_1_ and *d*_2_) are determined in the same way the pulse speed and width were determined in the no-stimulus case, which is by numerically solving a system of two transcendental equations. The first equation is given by *U*_3_(*d*_1_) = κ. The second equation is *U*_3_(*d*_2_) = κ if *d*_2_ < *x*_0_ + *d*_1_, else it is given by *U*_2_(*d*_2_) = κ. Figure 5 shows a plot of *d*_1_ (black curves) and *d*_2_ (gray curves) vs. the threshold κ. It can be seen that for a certain range of thresholds there exists more than one stable/unstable pair of pulses. Figure 6 shows the linear stability and the number of solutions for different combinations of stimulus speed (*v*) and strength (*I*_0_). The offset *x*_0_ = 3 and the corresponding spontaneous wave speed is *c* = 4. (Note that for smaller offsets *x*_0_ and thus smaller wave speeds *c*, one finds stimulus-locked waves for negative values of *v*). The stability of solutions in the presence of a stimulus is determined in much the same way as without a stimulus. We again define *u*(*x*, *t*) = U(ξ) + ϕ(ξ,t) and look at the behavior of the perturbations described by ϕ(ξ,t). Substituting into Equation (26), the stimulus term drops out when we perform the linearization, so that

$$\frac{\partial \varphi (\xi ,t)}{\partial t}=v\frac{\partial \varphi (\xi ,t)}{\partial \xi }-\varphi (\xi ,t)+\int_{-\infty }^{\infty }\text{​}w(\xi -y)\times \;F^{\prime }(U(y))\varphi (y,t)\text{ d}y.$$

Setting ϕ(ξ,t) = e^λ*t*^ϕ ultimately yields the spectral problem

(29) $$\begin{array}{c} \begin{array}{l} \lambda \varphi (\xi )\equiv ℒ\varphi (\xi )\\ \;=v\frac{\partial \varphi (\xi ,\;t)}{\partial \xi }-\varphi (\xi ,\;t)\\ \;+\frac{\left|v|\varphi (d_{1}\right)}{\left|\kappa -W(d_{1})-I(d_{1})\right|}w(\xi -d_{1})\\ \;+\frac{\left|v|\varphi (d_{2}\right)}{\left|\kappa -W(d_{2})-I(d_{2})\right|}w(\xi -d_{2}). \end{array} \end{array}$$

The corresponding Evans function is now

(30) $$ℰ(\lambda )=\left[K_{1}(w\;\times \;P_{\lambda })(0)-1\right]\left[K_{2}(w\;\times \;P_{\lambda })(0)-1\right]-K_{1}K_{2}(w\;\times \;P_{\lambda })(d_{2}-d_{1})(w\;\times \;P_{\lambda })(d_{1}-d_{2}),$$

where

$$K_{n}=\frac{\text{sgn}(v)}{\left|\kappa -W(d_{n})-I(d_{n})\right|},\;n=1,2,$$

and

$$P_{\lambda }(\xi )=H(-\text{sgn}(v)\xi ){\text{e}}^{(\lambda +1)\xi /v}.$$

**Figure 7:**
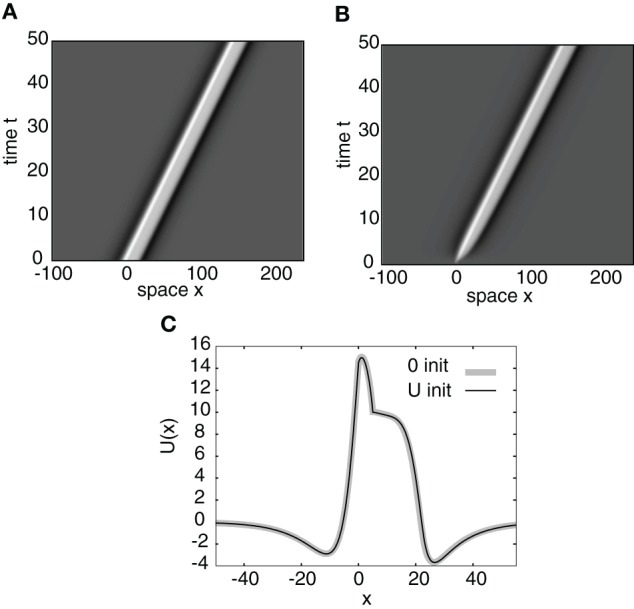
**Space-time plots of a stimulus-locked pulse for (A) *u*(*x*, 0) = U(*x*) and (B) *u*(*x*, 0) = 0.** Both initial conditions converge to the same wave profile in the large *t* limit as indicated in **(C)** for *t* = 50. Weight parameters as in Figure 2 and κ = 4. Stimulus parameters are *d* = 5, *I*_0_ = 8, and *v* = 3.

It is easy to establish as before that the residual spectrum is empty and that the continuous spectrum consists of a vertical line in the complex plane at Re(λ) = −1. So the stability is again determined only by the discrete spectrum, which consists of the zeros of the Evans function. Figure 7A shows an example of a numerical simulation of a stable stimulus-locked pulse solution of Equation (26) with the analytical pulse solution *U*(*x*) as an initial condition. Figure 7B shows the same simulation except with the zero initial condition *u*(*x*,0) = 0. It can be seen from Figure 7C that both initial conditions converge to the same pulse profile.

### Stochastic neural field

Several recent studies have considered stochastic versions of neural field equations that are based on a corresponding Langevin equation formulation (Brackley and Turner, [Bibr B7]; Hutt et al., 2008; Faugeras et al., 2009; Bressloff and Webber, 2012b). Motivated by these examples, we consider the following Langevin equation (or stochastic PDE) for the stochastic activity variable *U*(*x*, *t*), which is a rewriting of Equation (1) with *h*(*x*, *t*) given by Equation (5) for *I*_0_ = 0 and ϵ > 0:

(31) $$\text{d}U(x,t)=\left[-U(x,t)+\int_{-\infty }^{\infty }\text{​}w(x-y)F(U(y,t))\text{d}y\right]\text{d}t+\;\epsilon^{1/2}g(U(x,t))\text{d}W(x,t),$$

where d*W*(*x*, *t*) is an independent Wiener process with zero mean and correlation given by

(32) $$\langle \text{d}W(x,t)\text{d}W(x^{\prime },t^{\prime })\rangle =2C(|x-x^{\prime }|/\lambda )\delta (t-t^{\prime })dtdt^{\prime }.$$

Here λ is the spatial correlation length of the noise such that *C*(*x*/λ)→ δ(*x*) in the limit λ → 0, and ϵ determines the strength of the noise, which is assumed to be weak. For the sake of generality, we take the noise to be multiplicative rather than additive; however, the main results of the paper hold for both. Following standard formulations of Langevin equations (Gardiner, 2009), the multiplicative noise term is taken to be of Stratonovich form in the case of extrinsic noise. Note, however, that an alternative formulation of stochastic neural field theory has been developed in terms of a neural master equation (Buice and Cowan, 2007; Bressloff, 2009, 2010; Buice et al., 2010), in which the underlying deterministic equations are recovered in the thermodynamic limit *N*→ ∞, where *N* is a measure of the system size of each local population. In the case of large but finite *N*, a Kramers-Moyal expansion of the master equation yields a Langevin neural field equation with multiplicative noise of the Ito form Bressloff (2009, 2010). Multiplicative noise in the Stratonovich sense causes a shift in the speed and width of the pulse. This happens because 〈 *g*(*U*)dW〉≠ 0, even though 〈 d*W*〉 = 0. We can use Novikov's theorem (Novikov, 1965) to calculate the former average:

$$\epsilon^{1/2}\langle g(U)\text{d}W\rangle =\epsilon C(0)\langle g^{\prime }(U)g(U)\rangle \text{d}t.$$

The average can also be calculated by Fourier transforming Equation (31) and taking averages using the corresponding Fokker–Planck equation (Armero et al., 1998; Bressloff and Webber, 2012b). In the limit that λ approaches 0, we set *C*(0) → 1/Δx, where Δx is a lattice cut-off that can be identified with the spatial discretization step size in numerical simulations (Bressloff and Webber, 2012b). Following Ref. Armero et al. (1998), we rewrite Equation (31) so the fluctuating term has zero mean:

(33) $$\text{d}U(x,t)=\left[h(U(x,t))+\int_{-\infty }^{\infty }\text{​}w(x-y)F(U(y,t))\text{d}y\right]\text{d}t+\;\epsilon^{1/2}\text{d}R(U,x,t),$$

where

(34) $$h(U)=-U+\epsilon C(0)g^{\prime }(U)g(U)$$

and

(35) $$\text{d}R(U,x,t)=g(U)\text{d}W(x,t)-\epsilon^{1/2}C(0)g^{\prime }(U)g(U)\text{d}t.$$

The stochastic process *R* has zero mean and correlation

(36) $$\langle dR(U,x,t)dR(U,x^{\prime },t^{\prime })\rangle =\langle g(U(x,t))\text{d}W(x,t)g(U(x^{\prime },t^{\prime }))\text{d}W(x^{\prime },t^{\prime })\rangle +O(\epsilon^{1/2}).$$

#### Separation of time-scales

The effects of additive or multiplicative extrinsic noise on traveling waves can be analyzed using multiple time-scale methods originally developed for reaction-diffusion equations (Schimansky-Geier et al., 1983; de Pasquale et al., 1992; Armero et al., 1998; Sagues et al., 2007), which were recently extended to neural field equations in Ref. Bressloff and Webber (2012b). The main idea is to assume that the fluctuating term generates two distinct phenomena that occur on different time-scales: a diffusive-like displacement of the traveling wave from its uniformly translating position at long time-scales, and fluctuations in the wave profile around its instantaneous position at short time-scales. It is important to point out that, in contrast to traveling front solutions of scalar neural field equations (Bressloff and Webber, 2012b), we are now considering traveling pulse solutions. Thus in addition to the center-of-mass of the traveling pulse wave, which moves with speed *c* in the absence of noise, there is an additional degree of freedom corresponding to the “width” of the pulse. (In the case of a Heaviside rate function, the width Δ is determined by the threshold crossing points). For simplicity, we assume that the width of the wave is only weakly affected by the noise; this is consistent with what is found numerically. We now express the solution *U* of Equation (33) as a combination of a fixed wave profile *U*_0_ that is displaced by an amount Δ(*t*) from its uniformly translating position ξ = *x* − *c*_ϵ_*t*, where *c*_ϵ_ is a noise-dependent speed, and a time-dependent fluctuation Φ in the wave shape about its instantaneous position:

(37) $$U(x,t)=U_{0}(\xi -\Delta (t))+\epsilon^{1/2}\Phi (\xi -\Delta (t),t).$$

The wave profile *U*_0_ and associated wave speed/width *c*_ϵ_,Δ_ϵ_ are obtained by solving the modified deterministic equation

(38) $$-c_{\epsilon }\frac{\text{d}U_{0}(\xi )}{\text{d}\xi }-h(U_{0}(\xi ))=\int_{\infty }^{\infty }\text{​}w(\xi -\xi^{\prime })F(U_{0}(\xi^{\prime }))\text{d}\xi^{\prime }.$$

The results depend on ϵ due to the ϵ-dependence of *h*. Equation (38) is chosen so that that to leading order, the stochastic variable Δ(*t*) undergoes unbiased Brownian motion with a diffusion coefficient *D*(∈)=O(∈) (see below). The next step is to substitute the decomposition Equation (37) into (33) and expand to first order in O(∈^1/2^):

$$\begin{array}{l} -[c_{\varepsilon }+\dot{\Delta }]U_{0}^{\prime }(\xi_{\Delta })dt+\epsilon^{1/2}\left[d\Phi (\xi_{\Delta },t)-[c_{\epsilon }+\dot{\Delta }]\Phi^{\prime }(\xi_{\Delta },t)dt\right]\\ \;=h(U_{0}(\xi_{\Delta }))dt+\epsilon^{1/2}h^{\prime }(U_{0}(\xi_{\Delta }))\Phi (\xi_{\Delta },t)dt\\ \;+\int_{-\infty }^{\infty }w(\xi -\xi^{\prime })F(U_{0}(\xi_{\Delta }^{\prime }))d\xi^{\prime }dt\\ \;+\;\epsilon^{1/2}\int_{-\infty }^{\infty }w(\xi -\xi^{\prime })F^{\prime }(U_{0}(\xi_{\Delta }^{\prime }))\Phi (\xi_{\Delta }^{\prime },t)d\xi^{\prime }dt\\ \;+\;\epsilon^{1/2}dR(U_{0}(\xi_{\Delta }),\xi ,t)+O(\epsilon ). \end{array}$$

where we have set ξ_Δ_ = ξ − Δ(*t*) and ξ_Δ_' = ξ' − Δ(*t*). We now use Equation (38) for *U*_0_, after shifting ξ→ ξ − Δ(*t*), to eliminate terms and then divide through by $\sqrt{\epsilon }$. This gives the inhomogeneous equation to O(ϵ^1/2^)

(39) $$d\Phi (\xi_{\Delta },t)-\hat{L}\Phi (\xi_{\Delta },t)\text{d}t=\epsilon^{-\frac{1}{2}}{U^{\prime }}_{0}(\xi_{\Delta })d\Delta (t)+\;dR(U_{0}(\xi_{\Delta }),\xi ,t)$$

where the non-self-adjoint linear operator

(40) $$\begin{array}{c} \hat{L}A(\xi )\equiv c_{\epsilon }\frac{\text{d}A(\xi )}{\text{d}\xi }+h^{\prime }(U_{0}(\xi ))A(\xi )+\;\int_{-\infty }^{\infty }\text{​}w(\xi -\xi^{\prime })F^{\prime }(U_{0}(\xi^{\prime }))A(\xi^{\prime })\text{d}\xi^{\prime } \end{array}$$

is defined for all functions *A*(ξ) in ℒ_2_(ℝ). Note that for all terms in Equation (40) to be of the same order we have taken Δ(*t*) = O(ε^1/2^). It then follows that *U*_0_(ξ − Δ(*t*)) = U_0_(ξ) + O(ε^1/2^) etc., and Equation (39) reduces to

(41) $$d\Phi (\xi ,t)-\hat{L}\Phi (\xi ,t)\text{d}t=\epsilon^{-\frac{1}{2}}{U^{\prime }}_{0}(\xi )d\Delta (t)+dR_{u}(U_{0}(\xi ),\xi ,t)$$

If *U*_0_(ξ) were a traveling front solution of a neural field model with a symmetric, excitatory weight distribution *w*, then it could be proven that the operator $\hat{L}$ has a 1D null space spanned by *U*_0_'(ξ) (Ermentrout and McLeod, 1993). We will assume that such a result carries over to traveling pulse solutions of a neural field with *w* given by an asymmetric Mexican hat function; the fact that *U*_0_'(ξ) belongs to the null space follows immediately from differentiating Equation (38) with respect to ξ. We then have the solvability condition for the existence of a non-trivial bounded solution of Equation (41), namely, that the inhomogeneous part is orthogonal to all elements of the null space of the adjoint operator ${\hat{L}}^{*}$. The latter is defined with respect to the inner product

$$\int_{-\infty }^{\infty }\text{​}B(\xi )\hat{L}A(\xi )\text{ d}\xi =\int_{-\infty }^{\infty }\text{​}\left[{\hat{L}}^{*}B(\xi )\right]A(\xi )\text{ d}\xi .$$

Integrating by parts and using (Equation 14) leads to

(42) $$\begin{array}{c} {\hat{L}}^{*}B(\xi )=-c_{\epsilon }\frac{\text{d}B(\xi )}{\text{d}\xi }+h^{\prime }(U_{0}(\xi ))B(\xi )+\;F^{\prime }(U_{0}(\xi ))\int_{-\infty }^{\infty }w(\xi^{\prime }-\xi )B(\xi^{\prime })\text{d}\xi^{\prime }. \end{array}$$

We will assume that the null space of the adjoint operator ${\hat{L}}^{*}$ is also one-dimensional and is spanned by some yet to be determined function V(ξ). (In the case of a Heaviside firing function, we will determine the null space explicitly). Hence, we can write the solvability condition as

$$\int_{-\infty }^{\infty }V(\xi )\left[U_{0}^{\prime }(\xi )\text{d}\Delta (t)+\epsilon^{1/2}\text{d}R(U_{0}(\xi ),\xi ,t)\right]\text{ d}\xi =0.$$

which leads directly to the stochastic differential equation

$$\text{d}\Delta (t)=-\epsilon^{1/2}\frac{\int_{-\infty }^{\infty }\text{​}V(\xi )\text{d}R(U_{0},\xi ,t)\text{ d}\xi }{\int_{-\infty }^{\infty }\text{​}V(\xi )U_{0}^{\prime }(\xi )\text{d}\xi }.$$

Using the lowest order approximations *dR*(*U*_0_, ξ, *t*) = *g*(*U*_0_(ξ))*dW*(ξ, *t*), we deduce that [for Δ(0) = 0]

(43) $$\langle \Delta (t)\rangle =0,\;\langle \Delta {\left(t\right)}^{2}\rangle =2D(\varepsilon )t,$$

where *D*(ϵ) is the effective diffusivity

(44) $$D(\epsilon )=\epsilon \frac{\int_{-\infty }^{\infty }\text{​}V^{2}(\xi )g{\left(U_{0}(\xi )\right)}^{2}\text{ d}\xi }{{\left[\int_{-\infty }^{\infty }\text{​}V(\xi )U_{0}^{\prime }(\xi )\text{ d}\xi \right]}^{2}}.$$

#### Explicit results for heaviside rate function

In order to illustrate the above analysis, we consider a particular example where the mean speed *c*_ϵ_ and diffusion coefficient *D*(ϵ) can be calculated explicitly. That is, set *g*(*U*) = *g*_0_*U* for the multiplicative noise term and take *F*(*U*) = *H*(*u* − κ). (The constant *g*_0_ has units of $\sqrt{\text{length}/\text{time}}$). Note that the choice for *g*(*U*) can be interpreted physiologically in terms of an effective modification in the membrane time constant of neurons due to stochastic background synaptic activity (Bernander et al., 1991; Rapp et al., 1992; Bressloff, 1994). The deterministic Equation (38) for *U*_0_ then reduces to

(45) $$-\frac{\text{d}U_{0}(\xi )}{\text{d}\xi }+\Gamma (\epsilon )U_{0}(\xi )=\frac{1}{c_{\epsilon }}\int_{\infty }^{\infty }\text{​}w(\xi -\xi^{\prime })H(U_{0}(\xi^{\prime })-\kappa )\text{d}\xi^{\prime },$$

where Γ(ϵ) = (1 − ϵ*C*(0)*g*^2^_0_)/*c*_ϵ_. Hence,

(46) $$U(\xi )=\frac{e^{\Gamma \xi }}{c_{\epsilon }}\int_{\xi }^{\infty }W(\xi^{\prime }){\text{e}}^{-\Gamma \xi^{\prime }}\text{ d}\xi^{\prime }.$$

The deterministic pulse profile can be evaluated along identical lines to section “Neural Field Model of Direction Selectivity.” In order to calculate the diffusion coefficient, it is first necessary to determine the null vector V(ξ) of the adjoint linear operator ${\hat{L}}^{*}$. Substituting *F*(*U*) = *H*(*U* − κ) and *g*(*U*) = *g*_0_*U* into Equation (42) shows that

(47) $$\frac{dV(\xi )}{d\xi }+\Gamma (\epsilon )V(\xi )=\frac{\delta (\xi )}{c|U_{0}^{\prime }(0)|}\int_{-\infty }^{\infty }w(z)V(z)dz+\;\frac{\delta (\xi -\Delta )}{c|U_{0}^{\prime }(\Delta )|}\int_{-\infty }^{\infty }w(z-\Delta )V(z)dz.$$

Proceeding along similar lines to Bressloff (2001) and Kilpatrick et al. (2008), we make the ansatz that

(48) $$V(\xi )=AH(\xi ){\text{e}}^{-\Gamma \xi }+BH(\xi -\Delta ){\text{e}}^{-\Gamma (\xi -\Delta )}.$$

Substituting into Equation (47) shows that

$$A\;=\;\frac{1}{\left|U_{0}^{\prime }(0)\right|}[Ab(0)\;+\;Bb(\Delta )],\;B\;=\;\frac{1}{\left|U_{0}^{\prime }(\Delta )\right|}[Ab(-\Delta )\;+\;Bb(0)]$$

where

(49) $$b(z)\equiv \frac{1}{c}\int_{z}^{\infty }{\text{e}}^{-\Gamma (\xi^{\prime }-z)}w(\xi^{\prime })\text{ d}\xi^{\prime }.$$

Differentiating Equation (46) shows that *U*'(ξ) = *b*(ξ)−*b*(ξ − Δ), so that we obtain the vector equation

$$\left[\begin{array}{cc} \frac{b(0)}{b(0)-b(-\Delta )}-1 & \frac{b(\Delta )}{b(0)-b(-\Delta )}\\ \frac{b(-\Delta )}{b(0)-b(\Delta )} & \frac{b(0)}{b(0)-b(\Delta )}-1 \end{array}\right]\left[\begin{array}{l} A\\ B \end{array}\right]=0$$

The matrix has rank 1, confirming that the linear operator ${\hat{L}}^{*}$x has a 1D null-space. The latter is spanned by the function

(50) $$V(\xi )=b(\Delta )H(\xi ){\text{e}}^{-\Gamma \xi }-b(-\Delta )H(\xi -\Delta ){\text{e}}^{-\Gamma (\xi -\Delta )}.$$

**Figure 8:**
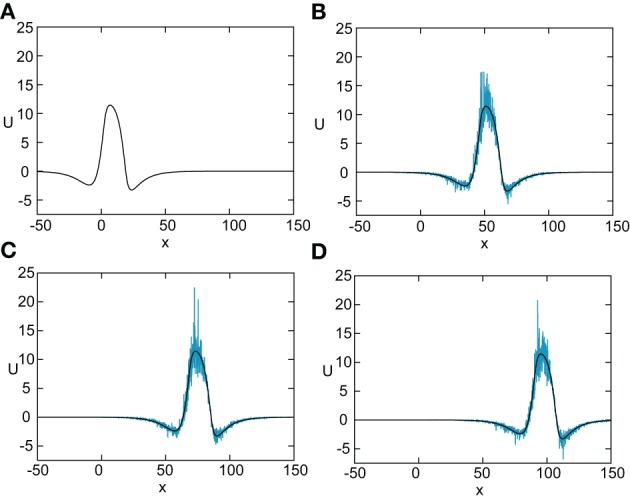
**Numerical simulation of freely propagating pulse solution of the stochastic neural field Equation (31) for a Heaviside rate function *F*(*U*) = *H*(*U*−κ) with κ = 4, and weight function (Equation 4) with *a*_*e*_ = 5, *a*_i_ = 1, σ_*e*_ = 0.42, σ_i_ = 0.1, and *x*_0_ = **3**.** The multiplicative noise is taken to be *g*(*U*) = *U*, the noise strength is ϵ = 0.005, and *C*(0) = 10. The wave profile is shown at successive times **(A)** *t* = 0 **(B)** *t* = 12 **(C)** *t* = 18, and **(D)** *t*=24, with the initial profile at *t*=0 given byequation *U*_0_. In numerical simulations we take the discrete space and time steps Δ*x* = 0.1, Δ*t* = 0.01. The deterministic part *U*_0_ of the stochastic wave is shown by the black curves.

**Figure 9:**
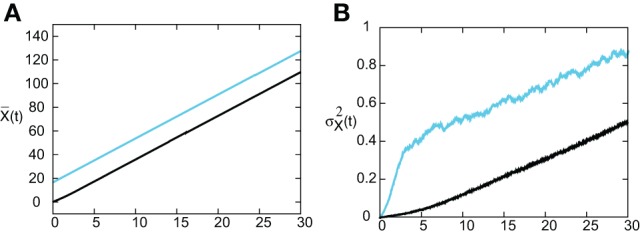
**(A)** Plot of mean position $\bar{X}(t)$ of leading (blue) and trailing (black) edges of pulse as a function of time *t* averaged over *N* = 4096 trials. **(B)** Corresponding plots of the variance σ^2^_*X*_(*t*). Same parameter values as Figure 8.

In Figure 8 we show the temporal evolution of a freely propagating stochastic traveling pulse, which is obtained by numerically solving the Langevin Equation (31) for *F*(*U*) = *H*(*U* − κ), *g*(*U*) = *U* and the asymmetric difference-of-exponentials (Equation 4). Note that the location of the stochastic wave appears to coincide with the underlying mean solution. However, over longer time-scales the wandering of the pulse about its mean position would be seen. In Figure 9 we plot the mean position $\bar{X}(t)$ and variance σ^2^_*X*_(*t*) of the leading and trailing edges of the pulse as a function of *t*. It can be seen that they all vary linearly with *t*, consistent with the assumption that there is a diffusive-like displacement of the center-of-mass of the pulse from its uniformly translating position at long time-scales. The slopes of these curves then determine the effective wave speed and diffusion coefficient according to $\bar{X}(t)$ ~ *c*_ε_*t* and σ^2^_*X*_(*t*)~ 2*D*(ε)*t*. Both the leading and trailing edges exhibit the same speeds and diffusivities (after a transient phase). The transients are caused by fluctuations in the mean width of the pulse which can be neglected for large *t*, where the difference in the size of fluctuations of the leading and trailing edges can be neglected.

**Figure 10:**
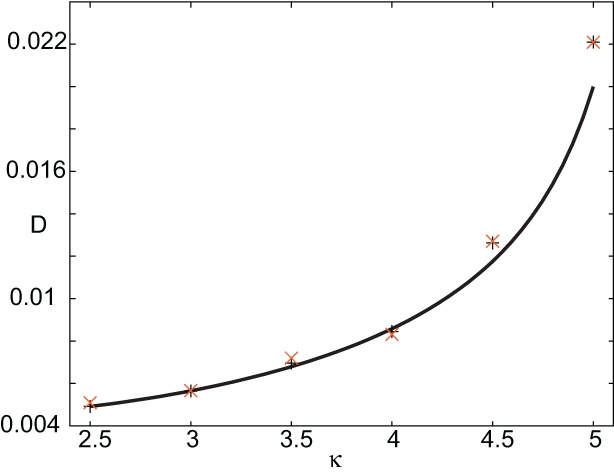
**Plot of diffusion coefficient *D*(ε) as a function of threshold κ.** Numerical results (“+” for leading edge,“X” for trailing edge) are obtained by averaging over *N*=4096 trials starting from the initial condition given by *U*_0_. Corresponding theoretical predictions (solid curves) for *D*(ε) are based on Equation (44). Other parameters as in Figure 8.

In order to find the mean location of the leading or trailing edge of the pulse as a function of time, we numerically carry out a large number of level set position measurements. That is, we determine the positions *X*_*a*_(*t*) such that *U*(*X*_a_(*t*),t) = a, for various level set values *a* and then define the mean location to be $\bar{X}(t)=E[X_{a}(t)]$, where the expectation is first taken with respect to the sampled values *a* and then averaged over *N* trials. The corresponding variance is given by $\sigma_{X}^{2}(t)=E[{\left(X_{a}(t)-\bar{X}(t)\right)}^{2}]$. In order to compare the numerical results with our theoretical analysis, we assume that *X*_*a*_(*t*) = Δ(*t*) + *c*_ϵ_*t* + *x*_*a*_(0) for each *a* on either the leading or trailing edge. It then follows that x$\bar{X}(t)$ = *c*_ϵ_*t* + *x*_*a*_(0) and σ^2^_*X*_(*t*) = 〈 Δ(*t*)^2^〉. In Figure 10 we plot the numerically estimated diffusion coefficient for various values of the threshold κ and compare these to the corresponding theoretical curves obtained using the above analysis. It can be seen that there is excellent agreement with our theoretical predictions. Finally, note that we can also use the level set data to estimate fluctuations in the width of the pulse. Suppose that *X*_*d*_(*t*) and *Y*_*d*_(*t*) denote the threshold crossing points of the leading and trailing edges of the pulse at time *t*. Then the stochastic width of the pulse can be defined according to *D*(*t*) = *X*_*d*_(*t*) − *Y*_*d*_(*t*). We find that after a transient phase, 〈*D*(*t*)^2^〉 − 〈*D*(*t*)〉^2^ « σ^2^_*X*_(*t*).

#### Stimulus-locked pulses

We now add a stimulus term *I* to the stochastic neural field Equation (33), that is

(51) $$\begin{array}{c} \text{d}U(x,t)=\left[h(U(x,t))+\int_{-\infty }^{\infty }\text{​}w(x-y)F(U(y,t))\text{ d}y\right]\text{d}t\\ +\;I(x-vt)dt+\epsilon^{1/2}\text{d}R(U,x,t), \end{array}$$

where the stimulus is again a rectangular wave of amplitude *I*_0_ and width *d*, moving with speed *v*. Here *h* and d*R* are defined by Equations (34) and (35). The stochastic activity variable is now decomposed according to Equation (37), with ξ = *x* − *vt*, and the modified deterministic equation

(52) $$-v\frac{\text{d}U_{0}}{\text{d}\xi }-h(U_{0}(\xi ))-I(\xi )=\int_{\infty }^{\infty }\text{​}w(\xi -\xi^{\prime })F(U_{0}(\xi^{\prime }))\text{ d}\xi^{\prime }.$$

Through a similar process as in the previous section, we expand to O(ϵ^1/2^) to obtain the inhomogeneous equation

(53) $$\begin{array}{c} d\Phi (\xi ,t)-\hat{L}\Phi (\xi ,t)\text{d}t=-\epsilon^{-1/2}\left[U_{0}^{\prime }(\xi )-I^{\prime }(\xi )\right]\text{d}\Delta (t)-\text{d}R(U_{0}(\xi ),\xi ,t)+O(\epsilon^{1/2}), \end{array}$$

where $\hat{L}$x is defined as in Equation (40) but with *c*_ϵ_ → v. The solvability condition is now

(54) $$\begin{array}{l} \int_{-\infty }^{\infty }V(\xi )[U_{0}^{\prime }(\xi )\text{d}\Delta (t)+I^{\prime }(\xi )d\Delta (t)+\epsilon^{1/2}\text{d}R(U_{0}(\xi ),\xi ,t)]\text{d}\xi =0. \end{array}$$

This can be manipulated to give, to leading order, the Ornstein–Uhlenbeck equation (Gardiner, 2009):

(55) $$\text{d}\Delta (t)+A\Delta (t)\text{d}t=\text{d}\hat{W}(t),$$

where

$$A=\frac{\int_{-\infty }^{\infty }\text{​}V(\xi )I^{\prime }(\xi )\text{d}\xi }{\int_{-\infty }^{\infty }\text{​}V(\xi )U_{0}^{\prime }(\xi )\text{d}\xi },$$

and

$$\text{d}\hat{W}(t)=-\epsilon^{1/2}g_{0}\frac{\int_{-\infty }^{\infty }\text{​}V(\xi )U_{0}(\xi )\text{d}W(\xi ,t)\text{ d}\xi }{\int_{-\infty }^{\infty }\text{​}V(\xi )U_{0}^{\prime }(\xi )\text{d}\xi }.$$

Solving the stochastic differential equation in Equation (55) and taking averages shows that 〈Δ(*t*)〉 = Δ(0)e^−*At*^ and

(56) $$\langle \Delta {\left(t\right)}^{2}\rangle -{\langle \Delta (t)\rangle }^{2}\approx \frac{D(\epsilon )}{A}\left[1-{\text{e}}^{-2At}\right],$$

where *D*(ϵ) is given by Equation (44) except for a modified null vector V(ξ). Thus the variance of Δ(*t*) approaches *D*(ϵ)/A in the large *t* limit.

**Figure 11:**
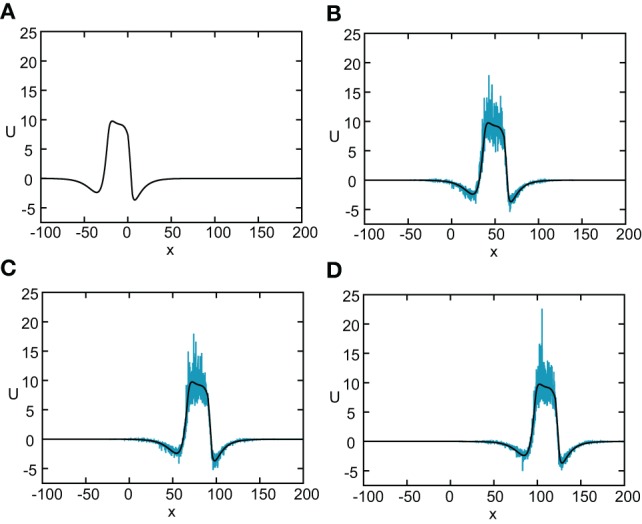
**Numerical simulation showing the propagation of a stimulus-locked pulse solution of the stochastic neural field Equation (51).** The external input is taken to be a square pulse with amplitude *I*_0_ = 5, width *d* = 5, and speed *v* = 5. All other parameters are as in Figure 8. The wave profile is shown at successive times **(A)** *t* = 0 **(B)** *t* = 6 **(C)** *t* = 12, and **(D)** *t* = 24, with the initial profile at *t* = 0 given by the solution *U*_0_. In numerical simulations we take the discrete space and time steps Δ*x* = 0.1,Δ*t* = 0.01.

**Figure 12:**
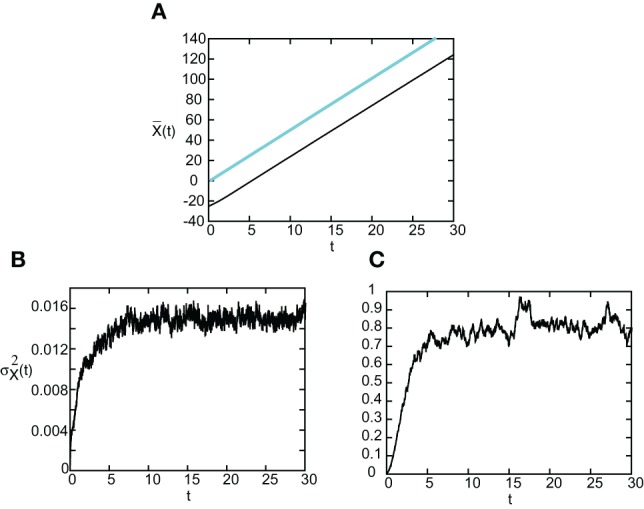
**(A)** Plot of mean position $\bar{X}(t)$ of leading (blue) and trailing (black) edges of stimulus-locked pulse as a function of time *t* averaged over *N* = 1000 trials. **(B,C)** Corresponding plots of the variance σ^2^_*X*_(*t*) of the leading and trailing edges. Same parameter values as Figure 11.

As in the case of freely propagating pulses, we can explicitly solve for V(ϵ) and thus calculate the diffusion coefficient *D*(ϵ) when *F*(*U*) = *H*(*U* − κ) and *g*(*U*) = *U*. Since the steps are similar to the previous case, we simply present our results here. In Figure 11 we show the temporal evolution of a single stimulus-locked front, which is obtained by numerically solving the Langevin Equation (51) for *F*(*U*) = *H*(*U* − κ), *g*(*U*) = *U* and the weight distribution (Equation 4). The external input is taken to be a square pulse of amplitude *I*_0_ = 5, width *d* = 5, and speed *v* = 5. Next we determine the mean $\bar{X}(t)$ and variance σ^2^_*X*_(*t*) of the position of the leading and trailing edges by averaging over level sets along identical lines to the freely-propagating case. The results are shown in Figure 12. It can be seen that, as predicted by the analysis, $\bar{X}(t)$ varies linearly with *t* with a slope equal to the stimulus speed *v* = 5. Moreover, the variance σ^2^_*X*_(*t*) approaches a constant value as *t* → ∞ for both the trailing and leading edges. Thus, we find that stimulus-locked pulses are much more robust to noise than freely propagating pulses, since the variance of the mean position of the leading and trailing edges saturate as *t*→ ∞. Consequently, stimulus locking persists in the presence of noise over most of the parameter range for which stimulus locking is predicted to occur. However, the trailing edge has an asymptotic variance that is at least an order of magnitude larger than the leading edge, which implies that fluctuations in the width of the pulse can no longer be neglected.

## Discussion

In this paper we have explored the effects of extrinsic noise on propagating pulses in a one-dimensional scalar neural field with asymmetric weights. Such a network has previously been proposed as a continuum model of direction selectivity. We have shown that the effects of noise on the wandering of the mean front position depends on properties of the underlying deterministic pulse. In the case of a freely propagating pulse, we find diffusive wandering with the mean square displacement growing linearly with time *t*. Moreover, in the large time limit, fluctuations in the width of the pulse can be neglected. On the other hand, if the pulse is locked to a moving pulse-like stimulus, then the wandering is described by an Ornstein–Uhlenbeck process and the mean square displacement saturates in the long time limit. However, we find that fluctuations in the pulse width can no longer be ignored.

In summary, this paper further illustrates how methods developed for studying wave propagation in stochastic PDEs can be adapted to study wave propagation in stochastic neural fields. As we have previously found for fronts, stimulus-locked waves are more robust to noise, which is a desirable property of a network performing some form of stimulus-processing such as direction selectivity.

### Conflict of interest statement

The authors declare that the research was conducted in the absence of any commercial or financial relationships that could be construed as a potential conflict of interest.
